# Segmentation of spinal rootlets across MRI contrasts with RootletSeg

**DOI:** 10.1038/s41598-026-49164-0

**Published:** 2026-05-02

**Authors:** Kateřina Krejčí, Jiří Chmelík, Sandrine Bédard, Falk Eippert, Ulrike Horn, Virginie Callot, Julien Cohen-Adad, Jan Valošek

**Affiliations:** 1 Department of Biomedical Engineering, FEEC, Brno University of Technology, Brno, Czechia; 2 NeuroPoly Lab, Institute of Biomedical Engineering, Polytechnique Montreal, Montreal, QC Canada; 3 Max Planck Research Group Pain Perception, Max Planck Institute for Human Cognitive and Brain Sciences, Leipzig, Germany; 4 Aix Marseille Univ, CNRS, CRMBM, Marseille, France; 5 APHM, CHU Timone, Pôle d’Imagerie Médicale, CEMEREM, Marseille, France; 6 Mila - Quebec AI Institute, Montreal, QC Canada; 7 Functional Neuroimaging Unit, CRIUGM, Université de Montréal, Montreal, QC Canada; 8 Centre de Recherche du CHU Sainte-Justine, Université de Montréal, Montreal, QC Canada; 9 Department of Neurosurgery, Faculty of Medicine and Dentistry, Palacký University Olomouc, Olomouc, Czechia; 10 Department of Neurology, Faculty of Medicine and Dentistry, Palacký University Olomouc, Olomouc, Czechia; 11 Spinal Cord Injury Center, Balgrist University Hospital, University of Zurich, Zurich, Switzerland; 12 Faculty of Medicine and Dentistry, Palacký University Olomouc, Hněvotínská 976/3, Nová Ulice, 779 00 Olomouc, Czechia

**Keywords:** Nerve rootlets, Spinal cord, Spinal levels, Segmentation, Deep learning, Anatomy, Medical research, Neurology, Neuroscience

## Abstract

Segmentation of spinal nerve rootlets is relevant for spinal level estimation, lesion classification, neuromodulation therapy, and group-level analyses. The aim of this study was to develop a deep learning method for the automatic segmentation of C2-T1 dorsal and ventral spinal nerve rootlets on various MRI scans. The study included MRI scans from two open-access and one private dataset, consisting of 3D isotropic 3T turbo spin echo T2-weighted (T2w) and 7T MP2RAGE (T1-weighted [T1w] INV1 and INV2, and UNIT1) MRI scans. A deep learning model, *RootletSeg*, was developed on 93 MRI scans from 50 healthy adults (mean age, 28.70 years ± 6.53 [SD]; 28 [56%] males, 22 [44%] females) and achieved a mean ± SD Dice score of 0.67 ± 0.09 for T1w-INV2, 0.65 ± 0.11 for UNIT1, 0.64 ± 0.08 for T2w, and 0.62 ± 0.10 for T1w-INV1 contrasts. RootletSeg accurately segmented C2-T1 spinal rootlets across MRI contrasts, enabling the determination of spinal levels directly from MRI scans. The method is open-source and can be used for a variety of downstream analyses.

**Supplementary Information:**

The online version contains supplementary material available at 10.1038/s41598-026-49164-0.

## Introduction

Spinal rootlets are bundles of nerve fibres forming the spinal nerves that connect the spinal cord to the peripheral parts of the body. The ability to estimate neurological spinal levels from nerve rootlets makes them relevant for spinal cord lesion classification^1,2^, neuromodulation therapy^3,4^, and fMRI group analysis^5–7^. Because directly identifying spinal rootlets on MRI scans is both challenging and time-consuming, spinal cord analyses typically rely on vertebral levels, defined using intervertebral discs, or they infer spinal levels from vertebral levels. This approach, however, is intrinsically limited as spinal levels are not necessarily aligned with vertebral bodies^5,8^, and there is considerable inter-individual variability between spinal and vertebral levels^8–13^. Spinal nerve rootlets, therefore, provide an anatomically more relevant and potentially more accurate method for determining spinal levels, which can, in turn, serve as an alternative coordinate system for spinal cord analyses, as opposed to the traditional vertebral-level approach based on intervertebral discs^5^. Recently, a method for automatic spinal rootlets segmentation was proposed, allowing for direct estimation of spinal levels from MRI data^14^. However, that method has three drawbacks: (1) it was developed solely on scans acquired at one field strength and with one contrast (i.e. 3T turbo spin echo [TSE] isotropic T2-weighted [T2w] scans), (2) it is restricted to dorsal rootlets only (ignoring ventral rootlets), and (3) it is restricted to a specific range of cervical levels (i.e. spinal levels C2-C8). Other studies aimed to identify nerve rootlets using diffusion MRI tractography^15^ or traced them manually on high-resolution scans^16^. An automatic rootlets segmentation method was recently proposed also for postmortem feline samples^17,18^.

In this work, we (1) extend the existing rootlet segmentation model by incorporating ventral rootlets, an additional spinal level (thoracic level T1), and additional MRI contrasts derived from the 7 T MP2RAGE sequence (T1-weighted [T1w] INV1 and INV2, and UNIT1); and (2) utilize the proposed segmentation model to investigate the correspondence between spinal and vertebral levels in a large cohort of 120 healthy participants. The segmentation method is open-source, implemented in the sct_deepseg function as part of Spinal Cord Toolbox (SCT)^19^ v7.0 and higher.

## Materials and methods

### Study design and participants

This retrospective study included scans from three MRI datasets of the cervical spinal cord (Suppl. Table 1): (i) 3T TSE isotropic T2w scans from the open-access single-site OpenNeuro ds004507 dataset^20^, (2) 3T TSE isotropic T2w scans from the open-access spine-generic multi-subject dataset^21^, and (3) 7T isotropic MP2RAGE scans from a private single-site dataset^22,23^. For more details on acquisition parameters,^20,21,22,24^. The inclusion and exclusion criteria are listed in the Supplementary material.

The experimental protocols for all datasets were approved by the relevant institutional ethics committees prior to data acquisition, specifically: the Comité d’éthique de la recherche du Regroupement Neuroimagerie Québec (OpenNeuro ds00450720 dataset^20^), local ethics committees of the participating institutions (spine-generic multi-subject dataset^21^) and by the Ethics Committee of the Medical Faculty of the University of Leipzig (private single-site dataset^22^). All datasets complied with all relevant ethical regulations, all experiments were performed in accordance with the Declaration of Helsinki and informed consent was obtained from each participant.

### Deep learning training protocol

The nerve rootlets segmentation model was developed using nnUNetv2, a self-configuring deep learning-based framework^25^. Details on data preprocessing and augmentation can be found in the Supplementary material.

**Fig. 1:**
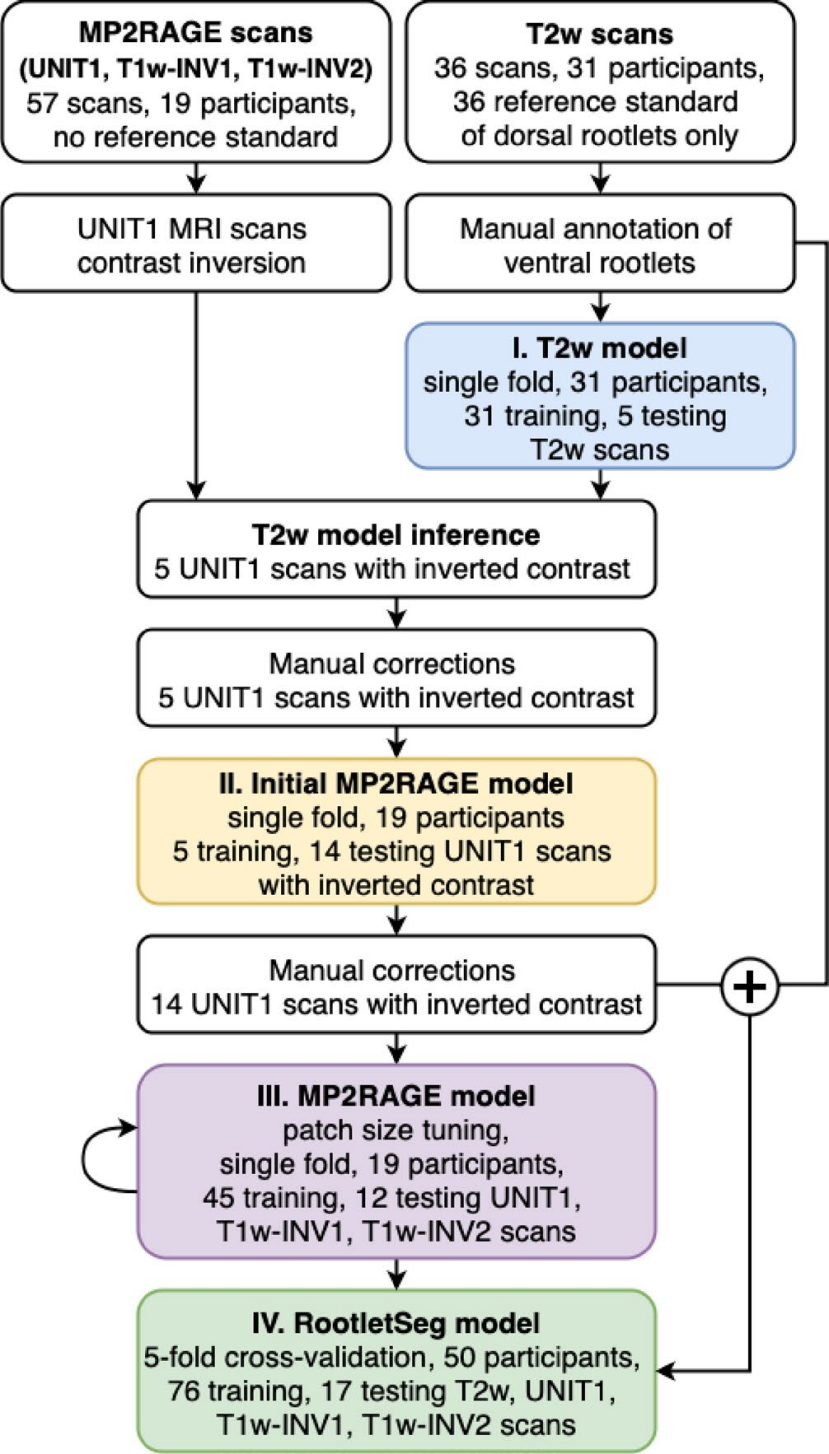
Overview of our model development. T2-weighted (T2w) scans with reference standards were first used to train the T2w model (I), which was then applied to MP2RAGE-UNIT1 scans with inverted contrast. The resulting outputs were manually corrected and served as reference standards for the training of the initial MP2RAGE model (II). This model was applied to additional UNIT1 scans, followed by another round of manual corrections. Next, the MP2RAGE model (III), using both the original and an increased patch size, was trained on all three MP2RAGE contrasts (T1w-INV1, T1w-INV2, and UNIT1). Finally, T2w and MP2RAGE scans and their corresponding reference standards were combined into a single dataset and used to train the *RootletSeg* model.

To facilitate the generation of reference standard rootlet labels, the existing segmentation model was applied^14^, followed by manual corrections by consensus of two raters (K.K. and J.V.) using the FSLeyes image viewer (version 1.12.1; University of Oxford) and the provided instructions [https://github.com/ivadomed/model-spinal-rootlets/issues/17]. We did not analyze the interrater variability, as a previous study reported a mean coefficient of variation of ≤ 1.45% in spinal level positions when using rootlet segmentations from different raters to estimate spinal levels^14^. Additionally, as the presence of C1 rootlets differs between individuals, they were not included in reference standard annotations and model training^12,14,26^. We trained the model in four stages by iteratively adding more MRI scans and contrasts in each stage (Fig. 1). Detailed training procedure is described in the Supplementary material. During model development, we observed that increasing the training patch size was necessary to successfully segment the C2 level, suggesting that a larger spatial context is important for accurate rootlet segmentation at this level. The final RootletSeg model configuration is included as a JSON file in the GitHub release (https://github.com/ivadomed/model-spinal-rootlets/releases/tag/r20250917). The model’s performance was evaluated using the Dice score and the 95th percentile Hausdorff distance (HD95)^27^, where a higher Dice score and a lower HD95 indicate better segmentation performance.

### Spinal-vertebral level analysis

The developed model was used to analyze the correspondence between spinal and vertebral levels. For MP2RAGE data, where each participant has three perfectly aligned contrasts, the T1w-INV2 contrast was used to avoid repeating the same participants multiple times. T1w-INV2 was selected due to its superior rootlet visibility and the highest *RootletSeg* performance among the MP2RAGE contrasts (see Results). From the OpenNeuro dataset, only participants with a neutral neck position were included to avoid repeating the same participants across different sessions. From the spine-generic dataset, MRI scans with clear difference in intensity values between the cerebrospinal fluid and nerve rootlets were selected. To be included in the analysis, each scan had to meet the following criteria: good visibility of spinal rootlets (no blurring and ghosting artifacts and good contrast between the rootlets and cerebrospinal fluid); coverage from the pontomedullary junction (PMJ) to the T1 vertebra; and available information on the participant’s height. Combining all three datasets, a total of 120 healthy participants were used.

**Fig. 2:**
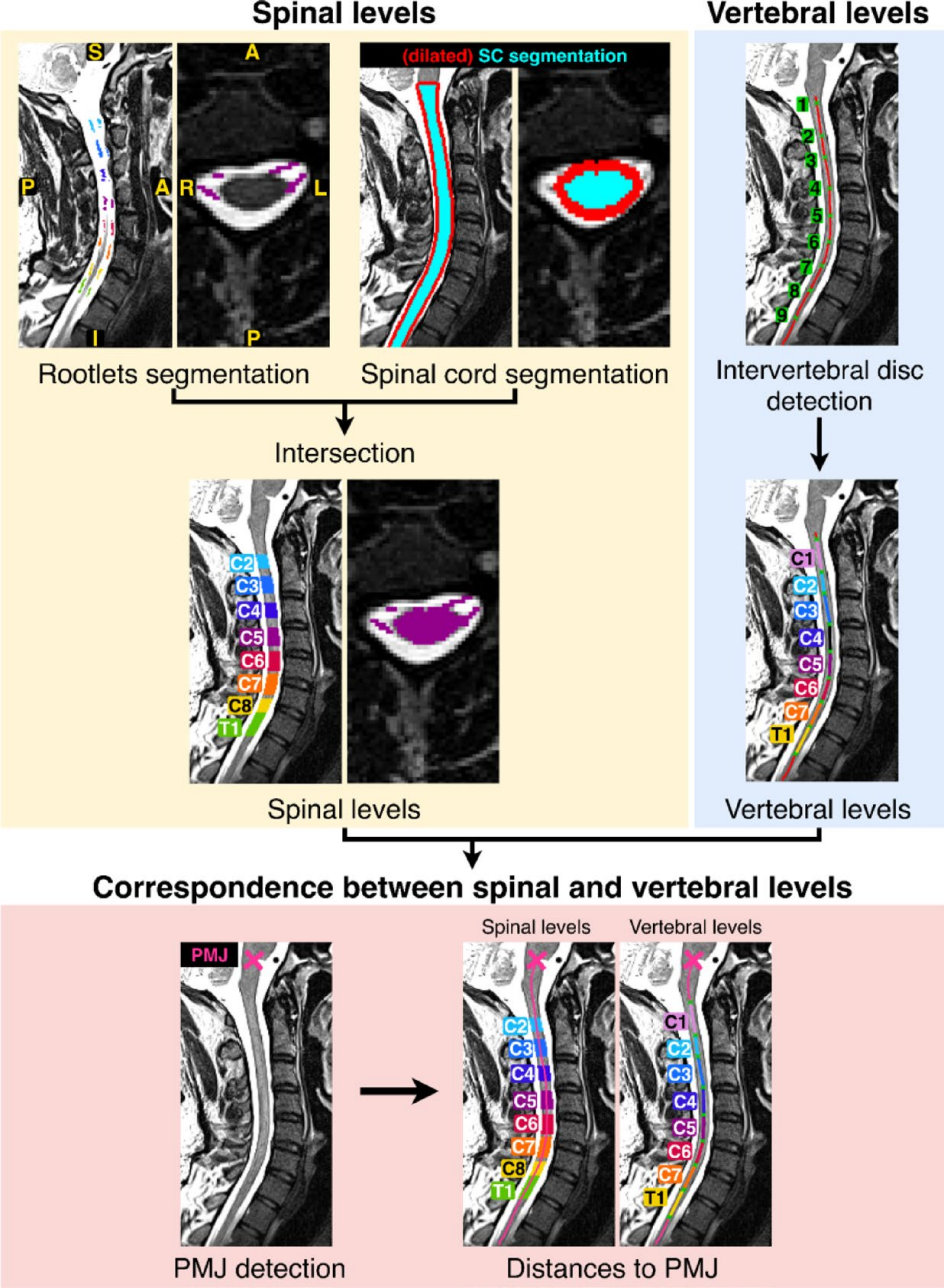
Spinal-vertebral levels correspondence. Analysis overview: the spinal cord and nerve rootlets were segmented, and the intervertebral discs and the pontomedullary junction (PMJ) were identified. Then, rootlets and intervertebral discs were used to obtain the spinal and vertebral levels, respectively. Distances from level midpoints to the PMJ were measured along the centerline.

Figure 2 illustrates the analysis pipeline for assessing spinal-vertebral level correspondence, performed using the SCT v7.0^19^. Spinal cord segmentations were available for all T2w MRI scans^20,21^, whereas the MP2RAGE scans were segmented using the contrast-agnostic model^28,29^. Spinal levels were estimated as an overlap between the spinal rootlets and the spinal cord segmentation dilated by 3 pixels^14^. The spinal cord centerline was extracted from the spinal cord segmentation, and the posterior tips of intervertebral discs^10^ were projected to the centerline. Vertebral levels were determined as the segments between adjacent intervertebral discs. PMJ was detected automatically^30^ and used as a reference point for further measurements.

The distances between the PMJ and the midpoints of the spinal and vertebral levels, as well as the level lengths, were measured along the spinal cord centerline. Spinal and vertebral level lengths were defined as the distance between the rostro-caudal slices of each level. To account for individual differences in body size, the distances were normalized by the participant’s height and then multiplied by the cohort’s median height to preserve millimetre units. The normalized distances were approximated by normal distributions using the probability density function to assess the overlap between spinal and vertebral levels. The correspondence between spinal and vertebral levels was evaluated using the Wilcoxon signed-rank test, the Bland–Altman analysis and root mean square distance (RMSD), separately for each level pair (e.g., vertebral level C2 and spinal level C3) across participants; Eq. 1 and 2 in the Supplementary material . We used a non-parametric variant of Bland–Altman analysis because the differences between spinal and vertebral level midpoint positions did not pass the Shapiro–Wilk normality test. *P* < 0.05 was considered statistically significant.

## Results

### Patient characteristics

A total of 134 healthy adult participants with 177 MRI scans from three datasets were included in this study. Participants were scanned across scanners from different manufacturers (Siemens, GE, Philips) with different field strengths (3T, 7T). For segmentation model development, we used 50 participants with 93 MRI scans (n = 12 OpenNeuro, n = 24 SpineGeneric and n = 57 MP2RAGE dataset) with 76 scans in the training set, and 17 scans in the testing set. For spinal-vertebral correspondence analysis, we used 120 MRI scans (n = 4 OpenNeuro, n = 105 SpineGeneric and n = 11 MP2RAGE) from 120 participants; 14 participants were not included because their MRI scans did not capture the PMJ and/or the scans did not capture the whole T1 vertebra.

### Segmentation model

**Fig. 3:**
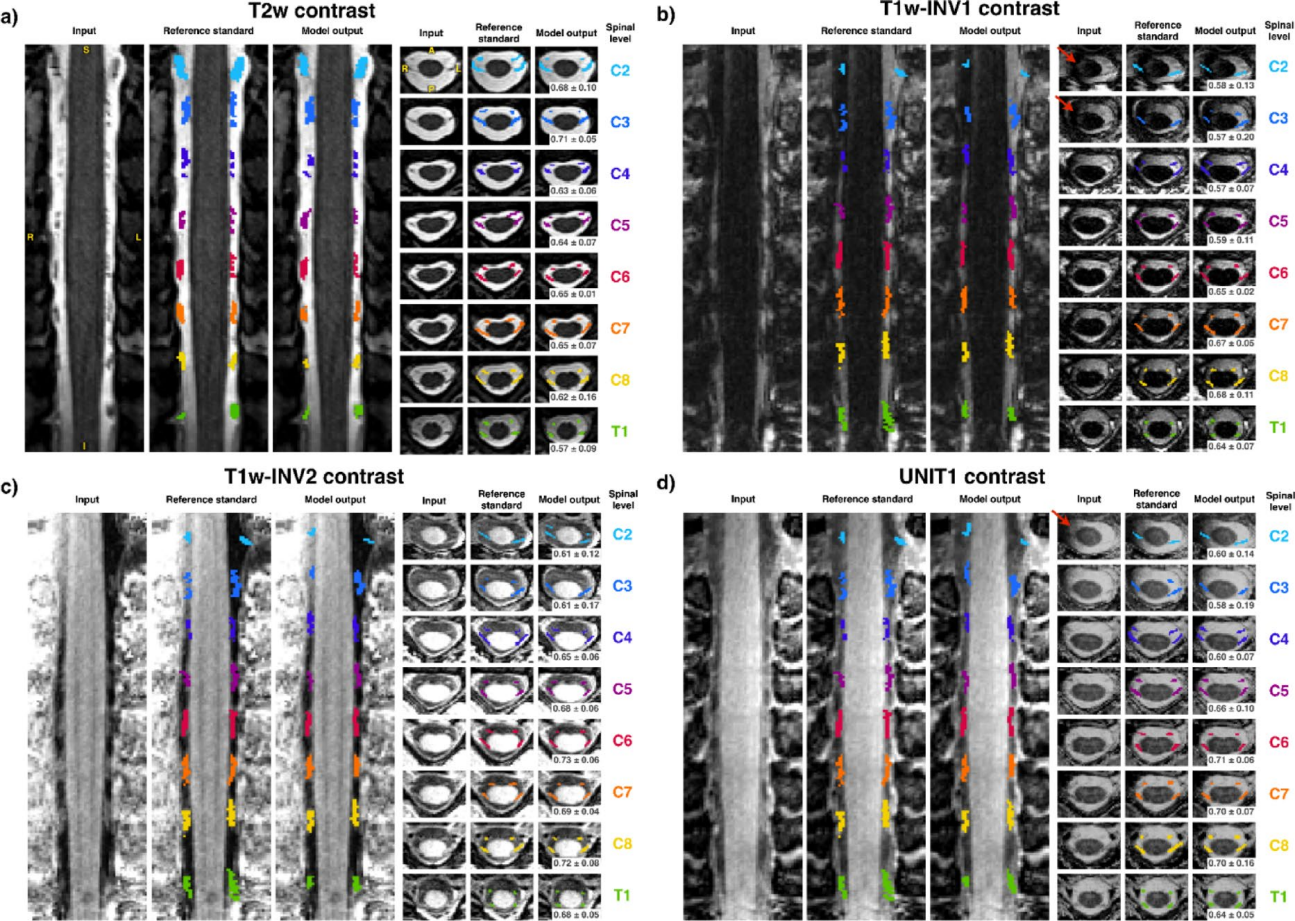
Coronal and axial views of representative rootlet segmentations. (**a**) Segmentations on a T2w MRI scan. (**b**) Segmentations on a T1w-INV1 MRI scan. (**c**) Segmentations on a T1w-INV2 MRI scan. (**d**) Segmentations on a UNIT1 MRI scan. The red arrows point to an artifact possibly caused by B1 + inhomogeneities. Rows represent individual rootlet levels from C2 to T1. Numbers represent the mean ± SD Dice score across participants for each spinal level compared to the reference standard labels.

**Fig. 4:**
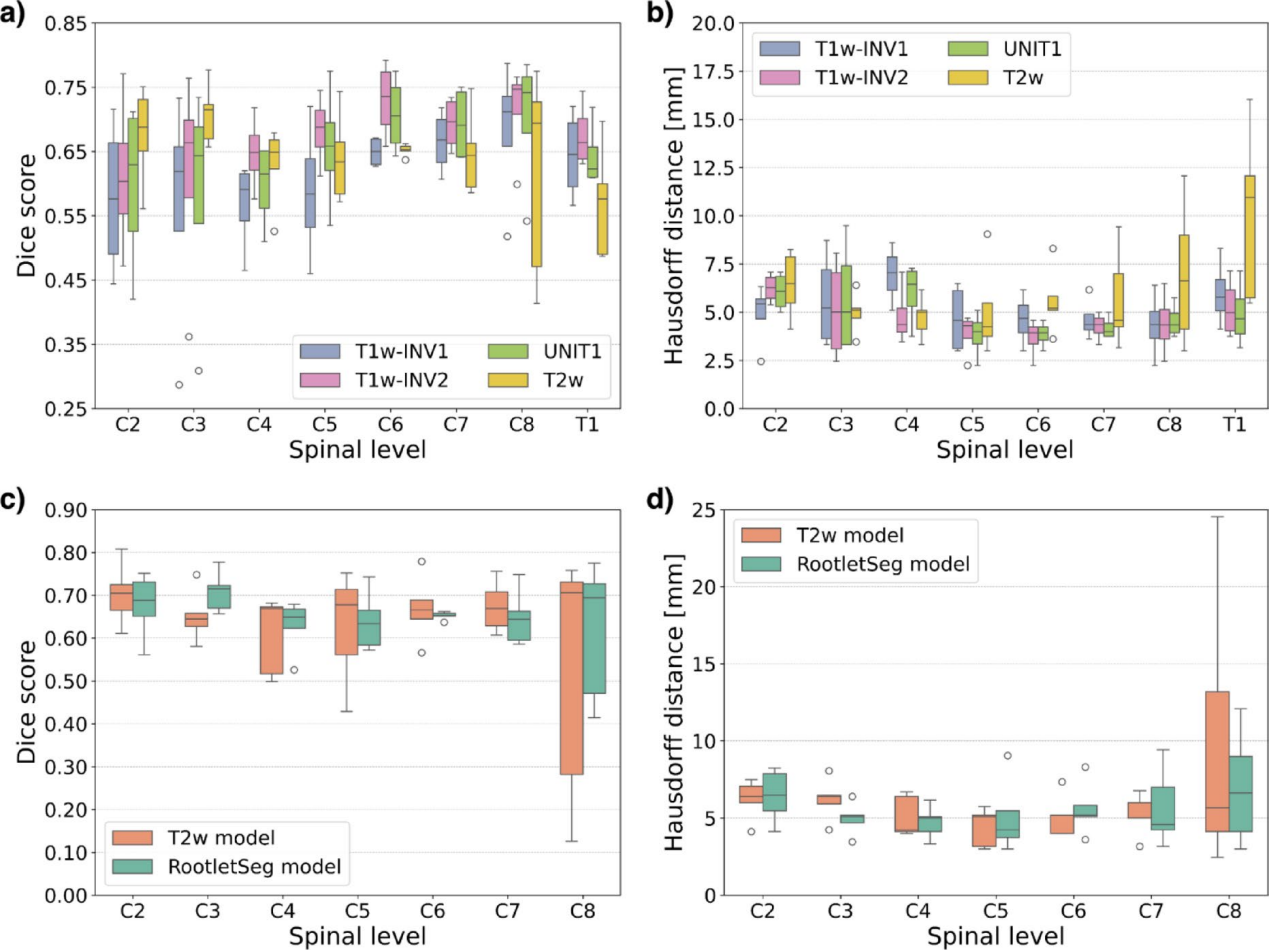
Quantitative performance of the *RootletSeg* model. (**a**, **b**) Dice score and HD95 computed on 17 testing images across different contrasts (n = 4 T1w-INV1, n = 4 T1w-INV2, n = 4 UNIT1 and n = 5 T2w). (**c**, **d**) Comparison of Dice score and HD95 between the intermediate single-contrast T2w model and the proposed *RootletSeg* model, on T2w MRI scans. Despite the *RootletSeg* model being trained on multiple contrasts, it performs similarly well compared to the T2w model. Note that the T2w model was developed only for spinal rootlets C2-C8; thus, T1 rootlets are not included in the comparison. The horizontal line within each box represents the median, and the box edges mark the 25% to 75% interquartile range. Whiskers extend 1.5 times the interquartile range, and the small black circles indicate outliers.

The *RootletSeg* nnUNetv2 3D model achieved an overall Dice score of 0.65 ± 0.10 and HD95 of 5.10 ± 1.82 mm (mean ± standard deviation [SD] across levels, participants, and contrasts). Lower Dice for the MP2RAGE scans at levels C2 and C3 was due to the presence of image artifacts (red arrows in Fig. 3), possibly caused by B1 + inhomogenities. Interestingly, despite the artifacts, the model was able to segment some rootlets at these levels, even though they were not included in the reference standard (compare the reference standard and model output for the T1w-INV2 MRI scan at the C2 level in Fig. 3). Dice scores for individual contrasts were (mean ± SD across levels and testing scans): 0.67 ± 0.09 for T1w-INV2, 0.65 ± 0.11 for UNIT1, 0.64 ± 0.08 for T2w, and 0.62 ± 0.10 for T1w-INV1 (Fig. 4a); and HD95 were: 4.70 ± 1.55 mm for T1w-INV2, 4.88 ± 1.60 mm for UNIT1, 6.20 ± 2.87 mm for T2w and 5.21 ± 1.75 mm for T1w-INV1 (Fig. 4b). Figure 4c,d show the Dice scores and HD95 values across levels on T2w MRI scans, in comparison with an existing model developed exclusively on T2w scans. *RootletSeg* demonstrated comparable results for rootlets C2-C8 to the T2w model (Dice of 0.65 ± 0.08 vs. 0.64 ± 0.08; HD95 of 5.65 ± 2.13 mm vs. 6.07 ± 3.75 mm). Level T1 was excluded from the Dice and HD95 calculation, as the T2w model was not trained at this level. The total inference runtime, measured on a single sample T2w scan, was 13.67 s on an NVIDIA GeForce RTX 3080 GPU 124 GB RAM and 639.63 s on an Apple M3 16 GB RAM.

### Spinal-vertebral level analysis

**Fig. 5:**
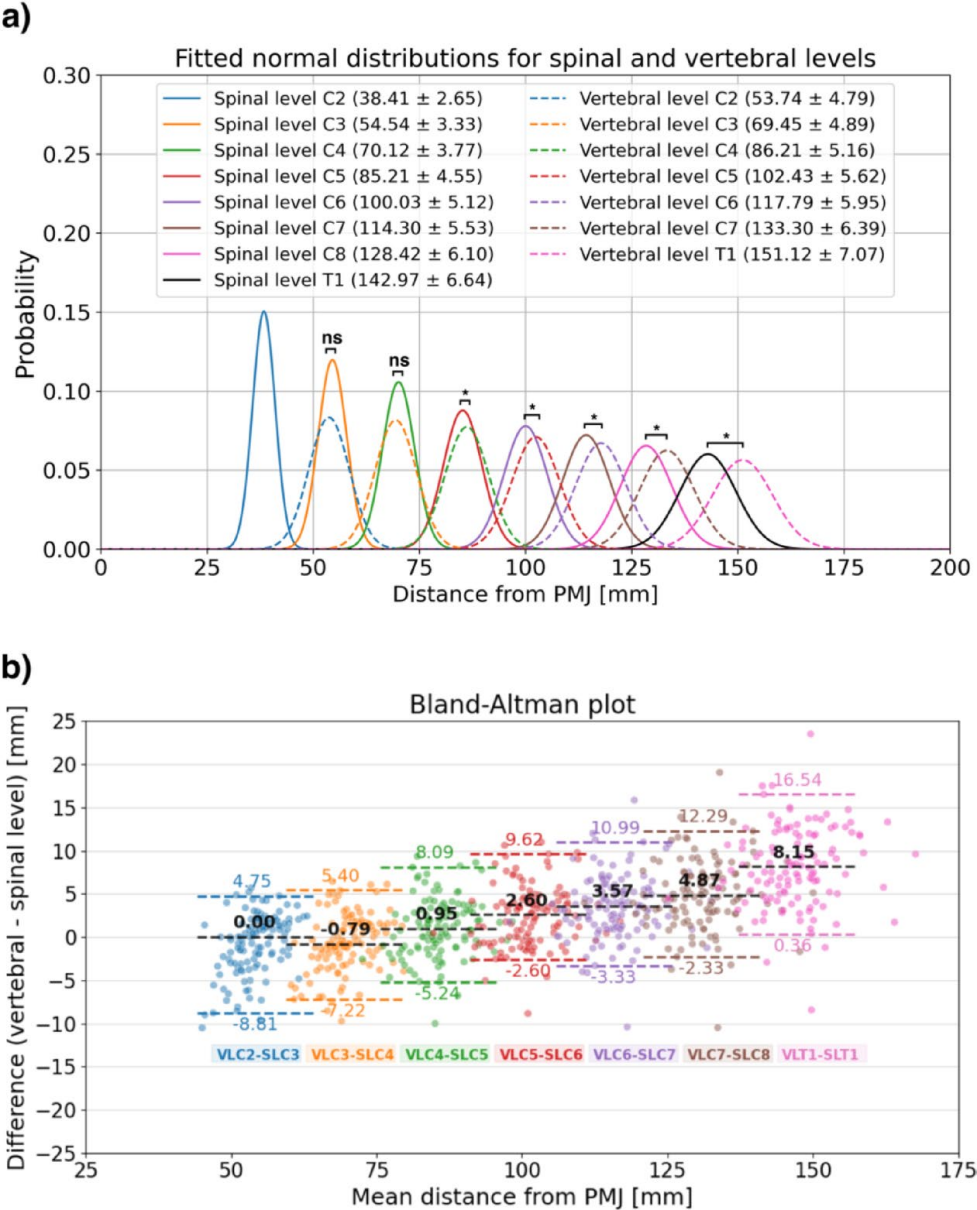
Spinal and vertebral level correspondence. (**a**) Spinal and vertebral level midpoints approximated by normal distributions, separately for each level. The midpoints were normalized by participants’ height and scaled by median height. Values in brackets represent mean ± SD distance to the pontomedullary junction (PMJ) in millimetres. Spinal levels are in solid lines, vertebral levels in dashed lines. Significance (Wilcoxon signed-rank test): *P < .05, *ns* not significant. Notice that the distribution for the spinal level C3 (solid orange) corresponds to the vertebral level C2 (dashed blue), while the distribution for the spinal level C8 (solid pink) is shifted cranially relative to the vertebral level C7 (dashed brown). We note that there are anatomically seven vertebral levels but eight spinal levels. (**b**) Bland–Altman plot. Black dashed lines show the median difference between distances from the PMJ to spinal and vertebral levels midpoints, and colored dashed lines show 2.5 and 97.5 percentiles. The points correspond to individual participants. VL = vertebral level; SL = spinal level.

Figure 5a illustrates the correspondence between spinal and vertebral levels. A gradually increasing *shift* along the rostrocaudal axis is apparent between the distributions of spinal and vertebral levels. For instance, the distribution for vertebral level C2 overlaps with that of spinal level C3, whereas vertebral level C7 is shifted caudally relative to spinal level C8. The Wilcoxon signed-rank test (performed for each spinal-vertebral level pair separately) revealed that this shift was statistically significant (*P* < 0.05) for all levels below the spinal level C5 and the vertebral level C4.

Figure 5b presents the Bland–Altman analysis comparing each pair of levels (e.g., vertebral level C2 vs. spinal level C3), based on the distance of the level midpoints from the PMJ, normalized by participant height. The Bland–Altman analysis quantitatively assessed a 0.00 mm bias term (median difference between spinal and vertebral level midpoint positions) between spinal level C3 and vertebral level C2. In contrast, the bias term is higher for the lower levels (up to 8.15 mm for spinal level T1 and vertebral level T1). Suppl. Table 2 shows the RMSD between spinal and vertebral level midpoints for each level pair. The RMSD value was lowest for spinal level C2 and vertebral level C3 (3.60 mm) and highest for spinal level T1 and vertebral level T1 (9.36 mm).

Suppl. Table 3 and Suppl. Table 4 show the rostro-caudal lengths of each spinal and vertebral level (mean ± SD across participants) in 120 healthy participants. Suppl. Table 3 includes a comparison with lengths reported in an MRI-based study^8^ and a post-mortem study^31^, and Suppl. Table 4 with a comparison to a post-mortem study^32^.

## Discussion

In this study, we (1) introduced *RootletSeg*, a deep learning model for segmentation of C2-T1 ventral and dorsal spinal nerve rootlets from T1w, T2w and MP2RAGE-UNIT1 3T and 7T MRI scans, which extended the previous rootlet segmentation model^14^ by incorporating ventral rootlets, an additional thoracic T1 spinal level, and additional 7T MP2RAGE-derived contrasts; and (2) investigate the correspondence between spinal and vertebral levels in a large cohort of 120 healthy participants. The segmentation model demonstrated stable performance across participants, MRI contrasts, and rootlet levels, thus facilitating the cumbersome and time-intensive manual rootlets annotation process. The analysis of spinal-vertebral level correspondence showed a gradually increasing shift in the rostro-caudal axis between spinal and vertebral levels and higher variability in level localization across participants with lower levels. The segmented nerve rootlets can be used as an alternative to commonly used intervertebral discs in various applications, including lesion classification based on neurological levels and registration of individual scans to a template for group-level analysis^5,6^.

As spinal nerve rootlets are fine structures with submillimeter dimensions and a complex anatomy that varies across participants, their segmentation is challenging even for expert raters. Despite these difficulties, the proposed model achieved a stable performance across four contrasts (T2w, T1w-INV1, T1w-INV2, and UNIT1) and different levels (C2-T1). For T2w data, the mean Dice was higher and HD95 was lower for upper cervical rootlets relative to lower rootlets (C2 mean Dice: 0.68 ± 0.08 vs. T1 mean Dice: 0.57 ± 0.09; C2 mean HD95: 6.44 ± 1.70 vs. T1 mean HD95: 6.97 ± 3.68), possibly due to the lower contrast between cerebrospinal fluid and rootlets and higher rootlets angulation in the lower levels^13^. Compared to the intermediate single-contrast T2w model, *RootletSeg* achieved a comparable Dice of 0.65 ± 0.08 vs. 0.64 ± 0.08 and a better HD95 of 5.65 ± 2.13 mm vs. 6.07 ± 3.75 mm, demonstrating no loss in performance while extending the model capabilities beyond T2w contrast. Although the reported segmentation performance may appear lower compared to other segmentation tasks, such as spinal cord^29,33^ and spinal canal^34^, where Dice scores commonly reach 0.9, we note that the relatively low Dice and high HD95 for rootlet segmentation is due to the distinct anatomy and size of rootlets compared to larger structures like the spinal cord. Spinal rootlets are small structures with complex three-dimensional anatomy, typically having only 2–3 voxel width in MRI scans with submillimeter in-plane resolution.

The analysis of spinal and vertebral level correspondence using the *RootletSeg* model showed that the distribution of spinal level C3 midpoint positions corresponds to that of vertebral level C2, and similarly for spinal level C4 and vertebral level C3. The correspondence became less consistent at lower levels, as indicated by a statistically significant shift between spinal and vertebral levels, leading to larger shifts between spinal and vertebral midpoint positions. Moreover, SD of the level midpoint distances from the PMJ increases in the lower levels (spinal level C2 SD = 2.65 mm vs. spinal level T1 SD = 6.64 mm), resulting in broader and flatter distributions, indicating increasing inter-subject variability in level positions. This is consistent with prior MRI and post-mortem reports^8,9^ and anatomical textbooks that neurological spinal levels are “*shifted*” relative to vertebral levels and that this shift increases at more caudal levels. Similar to a previous 3T MRI study^8^ that used manually defined landmarks, the Bland–Altman analysis showed higher variability in the position of lower levels across the participants. In our study, the analysis was extended to include levels C2 and T1, and we used 6 times more data. Additionally, we used participants’ height to normalize our measurements to take into account inter-subject variability. We also analyzed the level correspondence using RMSD, which confirmed a higher shift for more caudal levels by higher RMSD values compared to more cranial levels. The difference between the Bland–Altman and the RMSD analyses (e.g., Bland–Altman bias of 0.00 mm and RMSD of 3.60 mm for vertebral level C2 and spinal level C3) is due to methodological differences in the calculation—in the Bland–Altman analysis, we quantitatively considered the correspondence according to the median difference between vertebral-spinal midpoint positions, whereas the RMSD was calculated as the mean squared difference. A post-mortem study^9^, performed manually on 16 cadavers, found that spina-vertebral correspondence differs by one level in the C3 to C5 spinal region, i.e., vertebral level C2 corresponds to spinal level C3 and further increases in the lower levels up to two level differences. It needs to be noted that it is difficult to directly compare the findings from post-mortem studies with those in our in vivo MRI study due to the inherent characteristics of ex vivo measures, such as tissue shrinking due to post-fixation, and the altered shape of the excised spinal cord without the surrounding cerebrospinal fluid and dura.

Measured rostrocaudal spinal level lengths obtained in our study showed slightly higher SD compared to an MRI-based study^8^ and a post-mortem study^31^. This might likely be due to the larger cohort in our study capturing broader population variability, demographic differences, and differences in the acquisition protocol. Due to the lack of MRI studies on vertebral level lengths, our results were compared with a post-mortem study^32^, which measured vertebral levels from CT scans in six cadavers. For levels C3 to T1, our findings show similar results to the study. However, they measured the length of vertebral bodies, whereas our analysis was based on intervertebral disc positions. This different methodological approach likely contributes to the average difference of 2.1 mm observed across levels. Additionally, the small sample size (six cadavers in the study) may not adequately capture the population variability. Other factors that may account for the demographic differences and positional changes of the spine structures between living participants and cadavers.

This study had limitations. The proposed model was trained and tested solely on healthy participants in the C2-T1 region and only with isotropic MRI scans. Scans were selected based on overall image quality, and scans with extensive blurring and ghosting artifacts were excluded to allow for reliable reference standard labels creation. Extending the evaluation to participants with pathologies, including spinal cord injury or multiple sclerosis, and the thoraco-lumbar region would be valuable. However, this remains challenging due to the lower contrast typically present in pathological areas, such as a narrowed spinal canal. Additionally, in the thoraco-lumbar spinal cord, rootlets are more difficult to isolate because of their steeper angulation, reduced space within the spinal canal, potential image artifacts due to respiratory motion, and lower signal-to-noise ratio at 7T. These factors make it difficult to obtain reliable reference standard labels. For the spinal-vertebral correspondence analysis, we used the participants’ height to account for potential biological differences among individuals. The spinal cord or spine length could also be considered^11^, but MRI data typically does not cover the entire spine. We performed the level correspondence analysis on a population of healthy adults in the cervical region only, without distinguishing differences between males and females and between different age groups. Spinal–vertebral correspondence analysis relied on spinal cord and rootlet segmentations and may thus be influenced by segmentation accuracy. In the caudal region, the angulation of rootlets changes and a single axial image slice can contain rootlets from multiple spinal levels, making it difficult to reliably infer spinal levels using the intersection of rootlet and spinal cord segmentations. To address this increased complexity in these regions, it would be advantageous to propose a more robust method for obtaining spinal levels from rootlets segmentation^6^.

## Conclusion

In conclusion, this study presented *RootletSeg*, a deep learning model for C2-T1 spinal rootlets segmentation on T1w, T2w and MP2RAGE-UNIT1 MRI scans. The segmentation method is open-source, implemented in the sct_deepseg function as part of Spinal Cord Toolbox v7.0 and higher^19^. As the *RootletSeg* model allows for inferring the spinal levels directly from MRI, it can facilitate various downstream analyses, including lesion classification, neuromodulation therapy, and fMRI group analysis.

## Supplementary Information

Below is the link to the electronic supplementary material.

Supplementary Material 1

## Data Availability

The analysis scripts are open source and available at : The packaged and ready-to-use *RootletSeg* model can be applied to custom data via the sct_deepseg rootlets -i < MRI-scan > command as part of the Spinal Cord Toolbox (SCT) v7.0 and higher: https:/github.com/spinalcordtoolbox/spinalcordtoolbox/releases/tag/7.0. The data come from open-access datasets and can be accessed at https:/openneuro.org/datasets/ds004507/versions/1.1.1 and https:/github.com/spine-generic/data-multi-subject/tree/r20250314. The data from the private MP2RAGE dataset will be shared upon reasonable request.

## Abbreviations

PMJ: Pontomedullary junction
SD: Standard deviation
T1w: T1-weighted
T2w: T2-weighted

## References

[1] Mohajeri Moghaddam, S. & Bhatt, A. A. Location, length, and enhancement: Systematic approach to differentiating intramedullary spinal cord lesions. *Insights Imaging* **9**, 511–526 (2018). 29949034 10.1007/s13244-018-0608-3 PMC6108975

[2] Ahuja, C. S. et al. Traumatic spinal cord injury. *Nat. Rev. Dis. Primers*10.1038/nrdp.2017.18 (2017). 28447605 10.1038/nrdp.2017.18

[3] Vallejo, R. Neuromodulation of the cervical spinal cord in the treatment of chronic intractable neck and upper extremity pain: A case series and review of the literature. *Pain Physician* **2**(10), 305–311 (2007). 17387353

[4] Rowald, A. et al. Activity-dependent spinal cord neuromodulation rapidly restores trunk and leg motor functions after complete paralysis. *Nat. Med.* **28**, 260–271 (2022). 35132264 10.1038/s41591-021-01663-5

[5] Kinany, N. et al. Spinal cord fMRI: A new window into the central nervous system. *Neuroscientist* **29**, 715–731 (2023). 35822665 10.1177/10738584221101827 PMC10623605

[6] Bédard, S. et al. Rootlets-based registration to the spinal cord PAM50 template. *Imaging Neurosci.*10.1162/IMAG.a.123 (2025). 10.1162/IMAG.a.123 PMC12381661 40880898

[7] Schlienger, R. et al. Mapping human proprioceptive projections of upper limb muscles through spinal cord fMRI. *Hum. Brain Mapp.* **46**, e70386 (2025). 41123284 10.1002/hbm.70386 PMC12541885

[8] Cadotte, D. W. et al. Characterizing the location of spinal and vertebral levels in the human cervical spinal cord. *AJNR Am. J. Neuroradiol.* **36**, 803–810 (2015). 25523587 10.3174/ajnr.A4192 PMC7964298

[9] Kim, J. H. et al. Morphometric relationship between the cervicothoracic cord segments and vertebral bodies. *J. Korean Neurosurg. Soc.* **52**, 384–390 (2012). 23133729 10.3340/jkns.2012.52.4.384 PMC3488649

[10] Ullmann, E. et al. Automatic labeling of vertebral levels using a robust template-based approach. *Int. J. Biomed. Imaging* **2014**, 719520 (2014). 25132843 10.1155/2014/719520 PMC4123554

[11] Frostell, A. et al. A review of the segmental diameter of the healthy human spinal cord. *Front. Neurol.* **7**, 238 (2016). 28066322 10.3389/fneur.2016.00238 PMC5179522

[12] Diaz, E. & Morales, H. Spinal cord anatomy and clinical syndromes. *Semin. Ultrasound CT MR* **37**, 360–371 (2016). 27616310 10.1053/j.sult.2016.05.002

[13] Mendez, A. et al. Segment-specific orientation of the dorsal and ventral roots for precise therapeutic targeting of human spinal cord. *Mayo Clin. Proc.* **96**, 1426–1437 (2021). 33678411 10.1016/j.mayocp.2020.07.039

[14] Valošek, J. et al. Automatic segmentation of the spinal cord nerve rootlets. *Imaging Neurosci. (Camb.)* **2**, 1–14 (2024). 10.1162/imag_a_00218 PMC12272210 40800300

[15] Dauleac, C. et al. Full cervical cord tractography: A new method for clinical use. *Front. Neuroanat.* **16**, 993464 (2022). 36237419 10.3389/fnana.2022.993464 PMC9550930

[16] Liu, J. et al. An open-access lumbosacral spine MRI dataset with enhanced spinal nerve root structure resolution. *Sci. Data* **11**, 1131 (2024). 39406785 10.1038/s41597-024-03919-4 PMC11480038

[17] Fasse, A. et al. A novel CNN-based image segmentation pipeline for individualized feline spinal cord stimulation modeling. *J. Neural Eng.* **21**, 036032 (2024). 10.1088/1741-2552/ad4e6b 38772354

[18] Liang, L., Fasse, A., Damiani, A., et al. SpIC3D imaging: Spinal In-situ contrast 3D imaging. *bioRxiv*10.1101/2025.03.05.641747

[19] De Leener, B. et al. SCT: Spinal Cord Toolbox, an open-source software for processing spinal cord MRI data. *Neuroimage* **145**, 24–43 (2017). 27720818 10.1016/j.neuroimage.2016.10.009

[20] Bédard, S., Bouthillier, M. & Cohen-Adad, J. Pontomedullary junction as a reference for spinal cord cross-sectional area: Validation across neck positions. *Sci. Rep.* **13**, 13527 (2023). 37598229 10.1038/s41598-023-40731-3 PMC10439961

[21] Cohen-Adad, J. et al. Open-access quantitative MRI data of the spinal cord and reproducibility across participants, sites and manufacturers. *Sci. Data* **8**, 251 (2021). 34400655 10.1038/s41597-021-00941-8 PMC8368310

[22] Horn, U., Vannesjo, S. J., Gross-Weege, N., et al. Ultra-high-field fMRI reveals layer-specific responses in the human spinal cord. *bioRxiv*10.1101/2025.07.17.665316

[23] Massire, A. et al. High-resolution multi-parametric quantitative magnetic resonance imaging of the human cervical spinal cord at 7T. *Neuroimage* **143**, 58–69 (2016). 27574985 10.1016/j.neuroimage.2016.08.055

[24] Cohen-Adad, J. et al. Generic acquisition protocol for quantitative MRI of the spinal cord. *Nat. Protoc.* **16**, 4611–4632 (2021). 34400839 10.1038/s41596-021-00588-0 PMC8811488

[25] Isensee, F. et al. nnU-Net: A self-configuring method for deep learning-based biomedical image segmentation. *Nat. Methods* **18**, 203–211 (2021). 33288961 10.1038/s41592-020-01008-z

[26] Tubbs, R. S. et al. Clinical anatomy of the C1 dorsal root, ganglion, and ramus: A review and anatomical study. *Clin. Anat.* **20**, 624–627 (2007). 17330847 10.1002/ca.20472

[27] Maier-Hein, L. et al. Metrics reloaded: Recommendations for image analysis validation. *Nat. Methods* **21**, 195–212 (2024). 38347141 10.1038/s41592-023-02151-z PMC11182665

[28] Bédard, S. et al. Towards contrast-agnostic soft segmentation of the spinal cord. *Med. Image Anal.* **101**, 103473 (2025). 39874684 10.1016/j.media.2025.103473 PMC13110827

[29] Karthik, E. N., Sandrine, B., Jan, V., et al. Monitoring morphometric drift in lifelong learning segmentation of the spinal cord. *arXiv [csCV]* (2025). 10.1162/IMAG.a.1105 PMC12828353 41585468

[30] Gros, C. et al. Automatic spinal cord localization, robust to MRI contrasts using global curve optimization. *Med. Image Anal.* **44**, 215–227 (2018). 29288983 10.1016/j.media.2017.12.001

[31] Kobayashi, R. et al. A cadaveric study of the cervical nerve roots and spinal segments. *Eur. Spine J.* **24**, 2828–2831 (2015). 26084787 10.1007/s00586-015-4070-3

[32] Busscher, I. et al. Comparative anatomical dimensions of the complete human and porcine spine. *Eur. Spine J.* **19**, 1104–1114 (2010). 20186441 10.1007/s00586-010-1326-9 PMC2900026

[33] Gros, C. et al. Automatic segmentation of the spinal cord and intramedullary multiple sclerosis lesions with convolutional neural networks. *Neuroimage* **184**, 901–915 (2019). 30300751 10.1016/j.neuroimage.2018.09.081 PMC6759925

[34] Salmona, A. et al. Automated robust segmentation of the spinal canal on MRI. *Eur. J. Radiol. Artif. Intell.* **6**, 100075 (2026).

