# Supplementary material for: Segmentation of spinal rootlets across MRI contrasts with RootletSeg
Source: Sci Rep. 2026 May 2;16:20366. doi: 10.1038/s41598-026-49164-0 (PMC13332155; doi:10.1038/s41598-026-49164-0)
Supplement: Supplementary file 1 — Supplementary Material 1 [file 41598_2026_49164_MOESM1_ESM.pdf]

Supplementary material

Title

Segmentation of spinal rootlets across MRI contrasts with RootletSeg

Materials And Methods

Study Design and Participants

The scans were anonymized and defaced to remove all personally identifiable information (1,2) and organized according to the BIDS standard (3). The inclusion criteria were being a healthy participant without any known disease, age >18 years, and coverage of the cervical spine. Exclusion criteria were the presence of severe imaging artifacts or low contrast between the cerebrospinal fluid and nerve rootlets for multiple levels. Although the original datasets include more participants than listed in Suppl. Table 1, we selected scans based on overall scan quality (no blurring and ghosting artifacts) and sufficient contrast between the cerebrospinal fluid and nerve rootlets. Most exclusions were due to lower image quality, caused mainly by reduced spinal canal space or artifacts likely originating from B1+ inhomogenities (8 participants from the MP2RAGE dataset). Additional scans were excluded because the T1 spinal level was not included in the field of view (FOV) (11 participants), the pontomedullary junction (PMJ) was not in the FOV (7 participants), or height information was missing (2 participants). Because manual segmentation of nerve rootlets is difficult and time-consuming due to their complex three-dimensional anatomy (Suppl. Figure 1) and the scans' sub-millimetre resolution, only a subset of participants was included in model training (see the following sections for details).

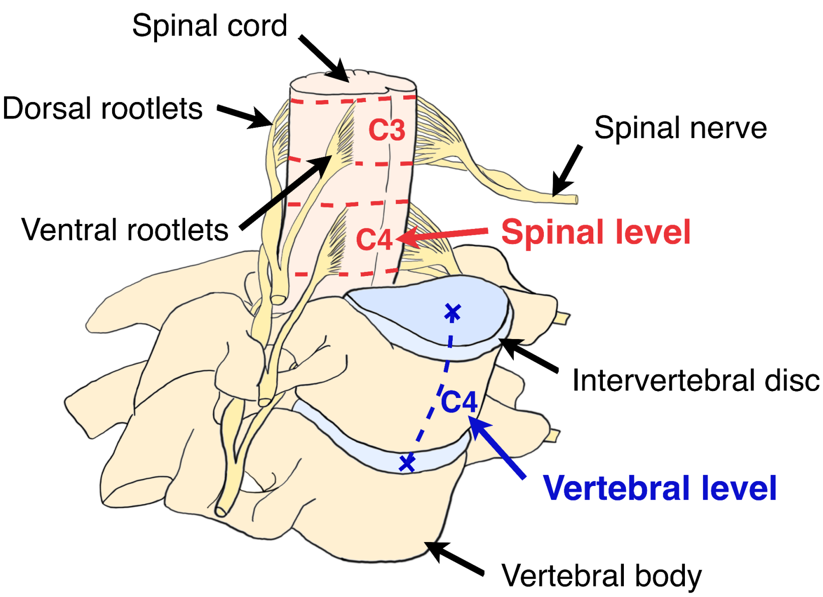

Supplementary Figure 1: Spinal rootlets and vertebral anatomy. Spinal levels are inferred from spinal rootlets, whereas vertebral levels are defined based on adjacent intervertebral discs. Adapted from (4) with permission from the publisher.

Deep Learning Training Protocol

Images and corresponding reference standard labels were reoriented to the Right-to-Left × Posterior-to-Anterior × Inferior-to-Superior (RPI) orientation to ensure consistency across the datasets. Preprocessing with the nnUNetv2 framework involved intensity z-score normalization and resampling into 0.7 × 0.7 × 0.7 mm resolution before model training. Random spatial transformations (translation, rotation, scaling), mirroring, Gaussian noise and Gaussian blur, brightness and contrast adjustment, Gamma transformation and low-resolution simulation were used for data augmentation.

We extended the T2w model to segment ventral rootlets by manually annotating them and retraining the model. Then, the T2w model was applied to MP2RAGE-UNIT1 scans. We inverted the contrast of MP2RAGE-UNIT1 scans to make their contrast closer to the T2w before applying the model. The model predictions were manually corrected and extended to include T1 rootlets. An initial MP2RAGE model was trained using five scans (MP2RAGE-UNIT1 with inverted contrast) and tested on the remaining 14 scans from the MP2RAGE dataset. The obtained segmentations were manually corrected to get a

reference standard for all 19 participants. Once all rootlet reference standards were visually inspected and corrected, they were split into training, validation, and testing sets. Because the MP2RAGE contrasts (T1w-INV1, T1w-INV2, and UNIT1) are inherently co-registered, a single reference standard was used for all three contrasts for each participant. To prevent information leakage between training, validation and testing sets, all MP2RAGE contrasts from each participant were assigned exclusively to one set.

Then, we trained the model on all three MP2RAGE contrasts (15 participants, resulting in 45 MRI scans) for 2000 epochs. The nnUNetv2 framework suggested a 3D architecture with the following parameters: 6 stages, instance normalization technique, Leaky ReLU activation function, batch size 2, patch size 128 × 96 × 192 (RPI), learning rate 0.01, Dice Loss and Cross-Entropy loss function and Stochastic Gradient Descent with Nesterov Momentum optimizer. Since training with this architecture setup was not successful at the C2 level, we tried another experiment with increased patch size in the superior-inferior direction to 352 (i.e., 128 × 96 × 352) so the model can capture a larger spatial context. With this increased patch size, the model successfully learned to segment the C2 level. The model training was consequently extended to a multi-contrast approach, adding data from T2w datasets (spine-generic and OpenNeuro). This model was trained on MP2RAGE and T2w MRI scans with the increased patch size (i.e., 128 × 96 × 352). A total of 76 scans (from 50 participants) for 2000 epochs in a 5-fold cross-validation approach were used for an 80/20% training/validation split. The "production" model, named RootletSeg, was trained on all 76 training data (100/0% training/validation split). The testing set (i.e., scans never used during the training or validation) included 17 MRI scans: 12 MP2RAGE (4 participants, each with 3 contrasts) and 5 T2w (3 from the SpineGeneric dataset and 2 from OpenNeuro). The same testing set was used across all five cross-validation folds to enable consistent evaluation of model performance across varied training/validation splits. The train–test split was performed at the participant level. Consequently, no participant overlap occurred between the training and testing sets across contrasts. The model was compared to the intermediate T2w single-contrast model.

Spinal-vertebral Levels Correspondence and Level Lengths

$$BlandAltman\ median\ =\ median(y_{vertebral\ (1)} - y_{spinal(1)},\ y_{vertebral\ (i+1)} - y_{spinal(i+1)},\ \dots\ ,\ y_{vertebral\ (n)} - y_{spinal(n)})$$

Equation 1

$y_{spinal(i)}$: distance between the PMJ and the spinal level midpoint

$y_{vertebral(i)}$: distance between the PMJ and the vertebral level midpoint

n … number of participants

$$RMSD = \sqrt{\sum_{i=1}^{n} \frac{(y_{vertebral(i)} - y_{spinal(i)})^2}{n}}$$

Equation 2

$y_{spinal(i)}$: distance between the PMJ and the spinal level midpoint

$y_{vertebral(i)}$: distance between the PMJ and the vertebral level midpoint

n: number of participants

Tables

RootletSeg model development

Variable	OpenNeuro ds004507*	spine-generic multi-subject	MP2RAGE**
**Participants**	7	24	19
**MRI scans**	12	24	57
**Sex**			
Male	5	12	11
Female	2	12	8
Age (y)	(22.57 ± 0.53)	(29.58 ± 6.53)	(29.84 ± 6.66)
**MRI scans in each set**			
Training set	10	21	45
Test set	2	3	12
**MRI manufacturer**			
Siemens	12	20	57
GE	0	4	0
**MRI field strength**			
3T	12	24	0
7T	0	0	57
**Sequence**	TSE	TSE	MP2RAGE
**Voxel size (mm)**	0.6 × 0.6 × 0.6	0.8 × 0.8 × 0.8***	0.7 × 0.7 × 0.7

Spinal-vertebral level correspondence analysis

Variable	OpenNeuro ds004507*	spine-generic multi-subject	MP2RAGE**
Participants	4	105	11
MRI scans	4	105	11
**Sex**			
Male	3	52	4
Female	1	53	7
Age (y)	(22.50 ± 0.58)	(29.70 ± 10.18)	(28.82 ± 6.03)
**MRI manufacturer**			
Siemens	4	76	11
GE	0	11	0
Philips	0	18	0
**MRI field strength**			
3T	4	105	0
7T	0	0	11
**Sequence**	TSE	TSE	MP2RAGE
**Voxel size (mm)**	0.6 × 0.6 × 0.6	0.8 × 0.8 × 0.8***	0.7 × 0.7 × 0.7

*The OpenNeuro ds004507 dataset included neutral, flexion and extension neck position sessions. Neutral and flexion sessions were used for the model development, and neutral session was used for spinal-vertebral correspondence analysis.
**The MP2RAGE dataset contained 3 co-registered MP2RAGE contrasts (T1w-INV1, T1w-INV2 and UNIT1) for each subject (in total 57 MRI scans for 19 subjects).
***Voxel size for 2 MRI scans was 0.8 × 0.5 × 0.5 mm

Supplementary Table 1: Characteristics of Study Participants

**Vertebral | Spinal level**	**RMSD [mm]**
C2 | C3	3.60
C3 | C4	3.35
C4 | C5	3.55
C5 | C6	4.21
C6 | C7	5.09
C7 | C8	6.33
T1 | T1	9.36

Supplementary Table 2: Root mean square distance (RMSD) between spinal and vertebral level midpoint distances to the pontomedullary junction (PMJ). Notice that RMSD is lower for cranial levels (i.e., RMSD of 3.60 mm between the vertebral level C2 and the spinal level C3) relative to caudal levels (i.e., RMSD of 9.36 mm between the vertebral level T1 and the spinal level T1). We note that there are anatomically seven vertebral levels but eight spinal levels.

**Spinal level**	**This work** (120 participants)	**Cadotte et al., 2015** (20 participants)	**Kobayashi et al., 2015** (11 participants)
C2	7.1 ± 2.2	-	-
C3	11.7 ± 2.5	10.5 ± 2.2	12.1 ± 1.2
C4	8.9 ± 2.0	9.9 ± 1.3	12.5 ± 1.1
C5	8.7 ± 1.7	10.5 ± 1.5	12.6 ± 2.8
C6	9.1 ± 1.6	9.7 ± 1.6	12.7 ± 1.6
C7	9.8 ± 1.7	9.4 ± 1.4	11.8 ± 1.6
C8	11.8 ± 2.5	9.6 ± 1.4	10.6 ± 1.6
T1	13.1 ± 3.7	-	-

Supplementary Table 3: Rostro-caudal lengths of individual spinal levels and results of MRI-based study (4) and post-mortem study (5). The table shows the mean rostro-caudal length ± SD in millimetres.

**Spinal level**	**This work** (120 participants)	**Busscher et al., 2010** (6 participants)
C2	14.9 ± 1.9	-
C3	17.0 ± 1.8	14.2 ± 0.7
C4	17.0 ± 1.6	14.5 ± 1.3
C5	15.9 ± 1.5	13.4 ± 1.1
C6	15.3 ± 1.7	14.0 ± 0.5
C7	16.2 ± 1.7	15.7 ± 0.9
T1	20.0 ± 1.8	17.3 ± 0.8

Supplementary Table 4: Rostro-caudal lengths of individual vertebral levels and results of the post-mortem study (6) (showing the mean rostro-caudal length ± SD in millimetres)

Supplementary References

1. Li X, Morgan PS, Ashburner J, Smith J, Rorden C. The first step for neuroimaging data analysis: DICOM to NIfTI conversion. J Neurosci Methods. Elsevier; 2016;264:47–56.

2. Gulban OF, Nielson D, Poldrack R, et al. poldracklab/pydeface: v2.0.0. Zenodo; 2019. doi: 10.5281/ZENODO.3524401.

3. Gorgolewski KJ, Auer T, Calhoun VD, et al. The brain imaging data structure, a format for organizing and describing outputs of neuroimaging experiments. Sci Data. 2016;3:160044.

4. Cadotte DW, Cadotte A, Cohen-Adad J, et al. Characterizing the location of spinal and vertebral levels in the human cervical spinal cord. AJNR Am J Neuroradiol. 2015;36(4):803–810.

5. Kobayashi R, Iizuka H, Nishinome M, Iizuka Y, Yorifuji H, Takagishi K. A cadaveric study of the cervical nerve roots and spinal segments. Eur Spine J. 2015;24(12):2828–2831.

6. Busscher I, Ploegmakers JJW, Verkerke GJ, Veldhuizen AG. Comparative anatomical dimensions of the complete human and porcine spine. Eur Spine J. 2010;19(7):1104–1114.
